# Mechanical and electronic response of monolayer chromium trihalides CrX_3_ (X = Cl, Br, I) under uniaxial strain

**DOI:** 10.1039/d5ra04294a

**Published:** 2025-09-08

**Authors:** Longlong Li, Maria Fyta

**Affiliations:** a Computational Biotechnology, RWTH Aachen University Worringerweg 3 52074 Aachen Germany l.li@biotec.rwth-aachen.de mfyta@biotec.rwth-aachen.de

## Abstract

Recent advances in two-dimensional (2D) magnetic materials have promoted significant progress in low-dimensional magnetism and its technological applications. Among them, atomically thin chromium trihalides (CrX_3_ with X = Cl, Br, and I) are among the most studied 2D magnets due to their unique magnetic properties. In this work, we employ density functional theory calculations to investigate the mechanical and electronic properties of CrX_3_ monolayers in the presence of in-plane uniaxial strain. We calculate the strain-dependent energetics, stress–strain relationships, electron localization functions, and electronic density of states for two distinct strain directions: zigzag and armchair. Our results show that the ferromagnetic phase remains the magnetic ground state over a wide range of strain in both zigzag and armchair directions. The mechanical response exhibits a distinct anisotropy between these two directions, as reflected by the maximum value in the stress–strain relationship. As the halogen atom X becomes heavier, the maximum stress decreases from 6.04 (6.40) GPa to 3.20 (3.33) GPa in the zigzag (armchair) direction. By analyzing the electron localization function, we show the strain-induced variations in bonding characteristics and their impact on the mechanical strength of the materials. We also observe a strain-induced energy splitting of the Cr-d and X-p orbitals in the electronic density of states, and a strain-induced band-gap transition (from indirect to direct) in the electronic band structure, leading to a reduction of the electronic band gap with a directional dependence on the applied strain. Our results provide fundamental insights into the effects of uniaxial strain on the mechanical and electronic properties of CrX_3_ monolayers, which are relevant for their potential use in strain-engineered electronics and spintronics.

## Abstract 2

The mechanical and electronic properties of ferromagnetic (FM) monolayer CrX3 (X = Cl, Br, I) under uniaxial strain reveal distinct tunable responses relevant to strain-engineered spintronics.

## Graphical abstract

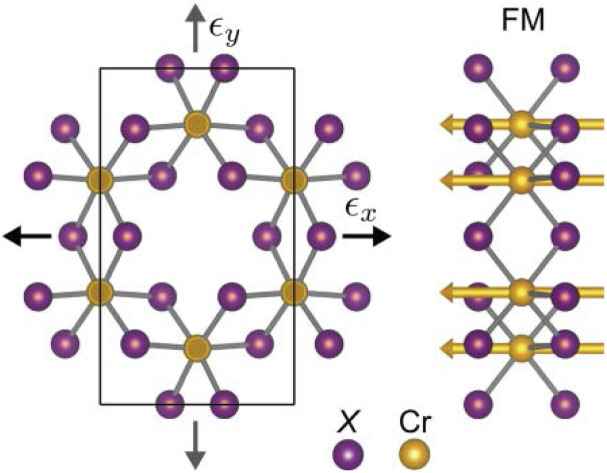

## Introduction

Two-dimensional (2D) magnetic materials have emerged as a fascinating combination of magnetic ordering and 2D materials, providing an exciting platform to explore fundamental magnetic phenomena and develop practical magnetic applications in the 2D limit.^1^ The ground-breaking discovery of intrinsic ferromagnetism in atomically thin CrI_3_ (ref. 2) and Cr_2_Ge_2_Te_6_ (ref. 3) in 2017 has triggered a revolution in the field of 2D magnetic materials. These 2D magnets exhibit unique magnetic properties that are not attainable in their bulk counterparts,^4^ including strong magnetic anisotropy and layer-dependent magnetic ordering, making them very attractive for low-power and high-efficiency spintronic applications.

The family of 2D magnets has expanded rapidly over recent years, within which a broad range of atomically thin materials with distinct magnetic properties have been discovered.^5^ For example, among the discovered 2D magnets, the following categories have been distinguished: CrI_3_,^2^ CrBr_3_,^6^ Cr_2_Ge_2_Te_6_ (ref. 3) are ferromagnetic semiconductors; CrCl_3_,^7^ CrTe_3_,^8^ FePS_3_ (ref. 9) are antiferromagnetic semiconductors; Fe_3_GeTe_2_,^10^ VSe_2_,^11^ GdTe_3_ (ref. 12) are ferromagnetic metals, and MnBi_2_Te_4_,^13^ MnBi_4_Te_7_ (ref. 14) are antiferromagnetic topological insulators. 2D magnetism can be measured using Raman spectroscopy,^15,16^ magneto-optic Kerr effect,^2,3^ magnetic circular dichroism,^17,18^ and anomalous Hall effect.^10,19^ The measured spectra can be interpreted using first-principles, Hamiltonian-based descriptions, and Monte Carlo simulations.^20–22^ Research progress has been made in understanding the roles of interlayer coupling, magnetic anisotropy, exchange interactions, and superexchange interactions in determining magnetic ordering and Curie temperature.^23–25^ The interplay between magnetism and other degrees of freedom, such as charge, spin, and valley, has led to the observation of numerous intriguing phenomena, including the quantum anomalous Hall effect,^13,26^ topological spin textures,^27,28^ and the anomalous valley Hall effect.^29,30^ Recent observations have also shown room-temperature ferromagnetism in 2D magnets, a crucial step towards practical applications.^10,11^ High-throughput computations and machine learning schemes have been used to identify promising new 2D magnets and guide experimental efforts.^31,32^

Atomically thin chromium trihalides (CrCl_3_, CrBr_3_, and CrI_3_), among the first and most important 2D magnets, have been widely investigated because of their highly tunable magnetic and electronic properties.^33,34^ In particular, biaxial strain has been shown to be an effective approach for tuning their magneto-electronic properties.^35–38^ However, the influence of uniaxial strain on these 2D magnets has received little attention. When compared to biaxial strain, uniaxial strain is anticipated to give rise to distinct anisotropies in the properties of these 2D magnets. In this work, we employ density functional theory (DFT) calculations to systematically investigate the mechanical and electronic properties of CrX_3_ (X = Cl, Br, I) monolayers in the presence of in-plane uniaxial strain. We calculate the strain-dependent energetics, stress–strain relationships, electron localization functions, and electronic density of states for two distinct strain directions: zigzag and armchair. We show that the ferromagnetic phase remains energetically favored over a wide strain range, indicating the robustness of ferromagnetism in these monolayers. The mechanical response exhibits a distinct dependence on the direction of the strain, as reflected in the ideal strength in the stress–strain relationship. By analyzing the electron localization function, we show the strain-induced variations in the bonding characteristics and their impact on the mechanical strength. We also observe the strain-induced splitting of the Cr-d and X-p orbitals in the electronic density of states, leading to a semiconductor-to-metal transition with directional dependence of transition strain. Our results provide fundamental insights into the effects of uniaxial strain on the mechanical and electronic properties of CrX_3_ monolayers, which are relevant for their potential applications in strain-engineered electronic and spintronic devices.

## Methodology

**Fig. 1:**
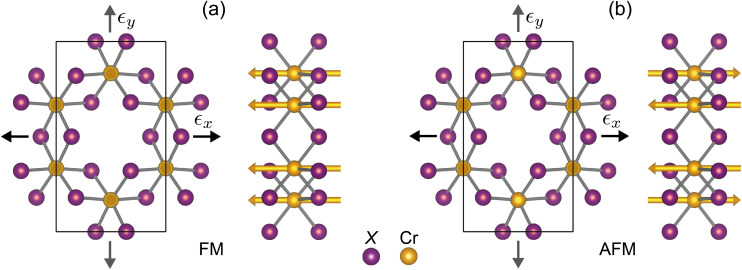
The lattice structure of CrX_3_ monolayer of both the top and side views with the (a) ferromagnetic (FM) and (b) antiferromagnetic (AFM) phases in the presence of an in-plane uniaxial strain *ε*_*x*_ or *ε*_*y*_, where the black rectangle is shown as the simulation cell and the strain *ε*_*i*_ is applied along the zigzag (*i* = *x*) or armchair (*i* = *y*) direction. Note that this structure is similar for all three CrX_3_ monolayers (X = Cl, Br, and I), but is different in their lattice vectors. The Cr and X atoms are shown in orange and purple, respectively.

Our DFT calculations were performed for monolayer chromium trihalides CrX_3_ (X = Cl, Br, I) using the projector-augmented wave method^39^ as implemented in the VASP code.^40^ The generalized gradient approximation (GGA) was used for the exchange-correlation functional as parameterized by Perdew, Burke, and Ernzerhof.^41^ Static electron correlation effects were included in the GGA + *U* approach^42^ employing *U*_eff_ = 2 eV for the X 3d orbitals, in line with previous work in the literature.^35,36^ A cutoff energy of 600 eV was used to truncate the plane wave expansion and a *Γ*-centered *k*-point mesh of 12 × 12 × 1 to sample the Brillouin zone (BZ). The lattice constants and the atomic coordinates were optimized until the forces on the atoms were less than 10^−3^ eV Å^−1^ and the change in the total energy was less than 10^−8^ eV. The vacuum layer was set to 20 Å to minimize the interaction between periodic simulation cells. The spin polarization due to the intrinsic magnetism was taken into account to reproduce the semiconducting nature of these monolayer systems. Two different magnetic configurations, *i.e.*, ferromagnetic (FM) and antiferromagnetic (AFM), were considered to evaluate the magnetic ground state by comparing their total energies. In the FM configuration, the magnetic moments were set to be parallel, while in the AFM configuration, the magnetic moments were set to be antiparallel. For both configurations, the magnetic moments were oriented in the off-plane direction. The lattice structure of monolayer CrX_3_ and the two magnetic configurations (FM and AFM) are illustrated in Fig. 1.

To explore the effects of strain, we considered the in-plane uniaxial tensile strain applied along the *x* and *y* directions, as illustrated in Fig. 1. This straining mode is typically used in theoretical investigations of strained materials^43–45^ to resemble the uniaxial tensile loading in the experiments^46^ and can resemble and compare to experimental conditions.^47^ The uniaxial strain was defined as *ε*_*i*_ = (*a*_*ε*_^*i*^ − *a*_0_^*i*^)/*a*_*i*_^0^ with *a*_*ε*_^*i*^ and *a*_0_^*i*^ being the strained and unstrained lattice constants along *i* direction (*i* = *x*, *y*). In our DFT calculations, the lattice structure of monolayer CrX_3_ was fully relaxed perpendicular to the strain direction to account for Poisson's effect. Specifically, for each applied uniaxial strain, the transverse lattice parameter was optimized to ensure minimal stress (close to zero) in the perpendicular direction.

In order to assess the dynamical stability of monolayer CrX_3_ (X = Cl, Br, I) in the absence and presence of uniaxial strain, density functional perturbation theory (DFPT)^48^ was used to calculate their phonon dispersions using the Phonopy code.^49^ For the DFPT phonon calculations, the hexagonal and rectangular unit cells were adopted in the absence and presence of uniaxial strain, respectively. To further inspect their thermal stability in the absence and presence of uniaxial strain, *ab initio* molecular dynamics (AIMD) simulations were performed on a 3 × 3 supercell at a temperature of 500 K for a simulation period of 12 ps with a time step of 2 fs.

## Results and discussion

**Fig. 2:**
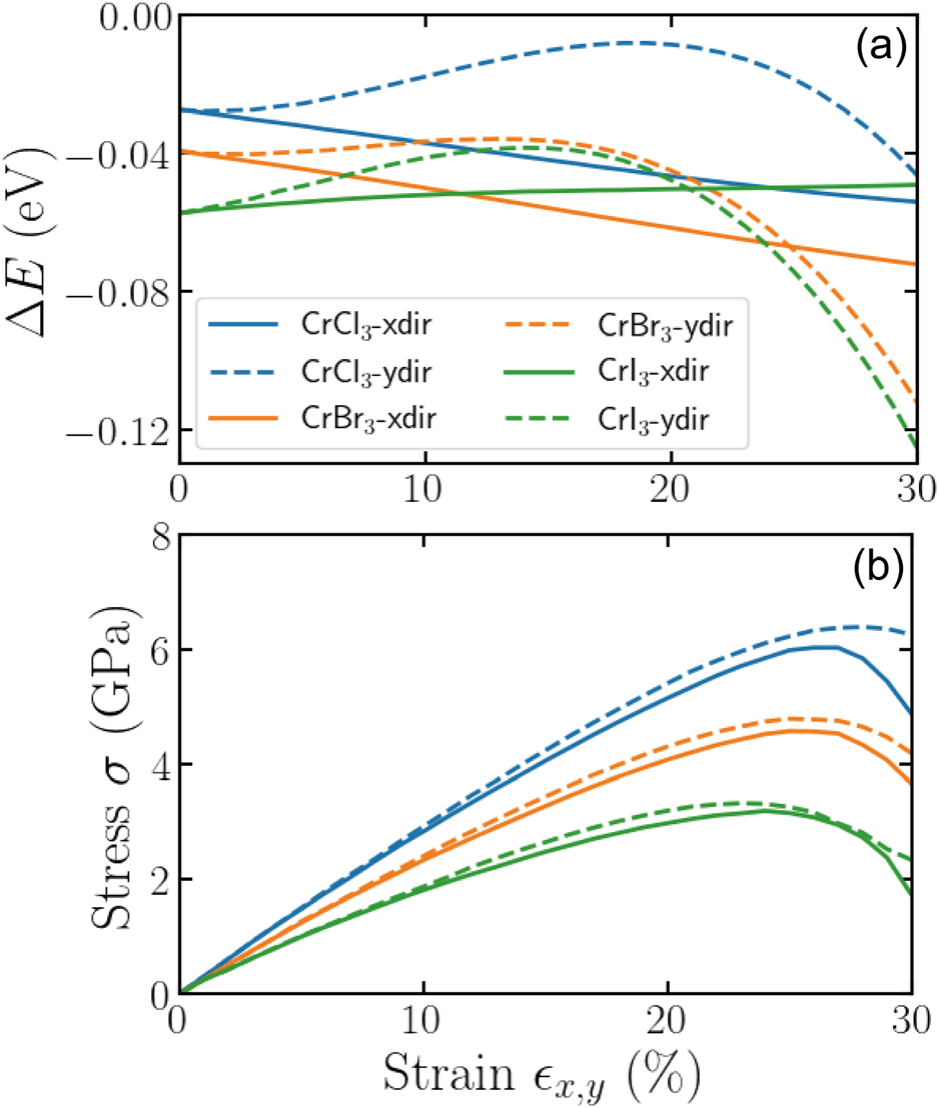
(a) The strain-dependent energetics and (b) the stress–strain relationships of CrX_3_ monolayers (X = Cl, Br, I) for both zigzag (*x*) and armchair (*y*) directions, denoted as *x* – dir and *y* – dir in the legend, respectively. Here, Δ*E* is defined as the energy difference of the AFM from the FM phase.

We begin with the analysis on the effects of uniaxial strain on the magnetic phase energetics and mechanical response of CrX_3_ (X = Cl, Br, I) monolayers as depicted in Fig. 2. The strain is applied along the zigzag (*x*) and armchair (*y*) directions. In Fig. 2(a), the energy difference (Δ*E*) between the FM and AFM phases is shown as a function of strain, with negative values indicating the energetic preference for the FM phase by the ground state. Across the three monolayers and for both strain directions, Δ*E* remains negative up to 30% strain, confirming that the FM phase is energetically favored by the ground state over the AFM phase. Unless otherwise specified, all results and discussions presented in the following will refer to the FM phase as this was confirmed as the magnetic ground state of the CrX_3_.

In Fig. 2(b), the mechanical response of the three monolayers is shown through the respective stress–strain curves. Apparently, the stress–strain relation exhibits a first quasi-linear and then nonlinear increase in stress with increasing strain, reaching a maximum value before decreasing with further increasing strain. Overall, all materials are plastically deformed and show a more ductile behavior. This is less evident for the strain along the *x* (zigzag) direction, which reveals a more brittle-like behavior. All studied monolayers can sustain a strain of up to roughly 30%. The maximum stress follows the trend CrCl_3_ > CrBr_3_ > CrI_3_, demonstrating that CrCl_3_ is the most rigid to mechanical deformation, while CrI_3_ is the most mechanically flexible. As the halogen atom X becomes heavier, the calculated maximum strength of the monolayers decreases from 6.04 (6.40) GPa to 3.20 (3.33) GPa in the zigzag (armchair) direction. The stress values are also consistently higher for strain applied along the armchair direction than the zigzag direction, revealing a distinct mechanical anisotropy in these monolayers. The fact that stress builds up more in the armchair direction suggests that the monolayers are mechanically stronger along this direction, indicating they require more force to deform when compared to the zigzag direction. These differences arise from variations in the atomic bonding arrangements and the distribution of interatomic forces in each direction.

Interestingly, while stress increases with strain in both directions, the maximum stress is reached at a lower strain value for the zigzag direction, indicating that CrX_3_ monolayers may reach mechanical failure earlier when stretched along this direction. This suggests that while these monolayers exhibit good mechanical flexibility overall, their ability to withstand strain depends sensitively on the direction of strain, which is important for designing strain-engineered applications. Furthermore, the observed decrease in stress after reaching its maximum indicates that structural instability or mechanical failure may occur beyond this threshold, particularly for CrI_3_, which is already the most flexible among the three. These findings highlight the potential of CrX_3_ monolayers as strain-tunable materials with robust FM stability, making them attractive for potential applications in mechanically flexible and strain-engineered spintronic devices.

**Fig. 3:**
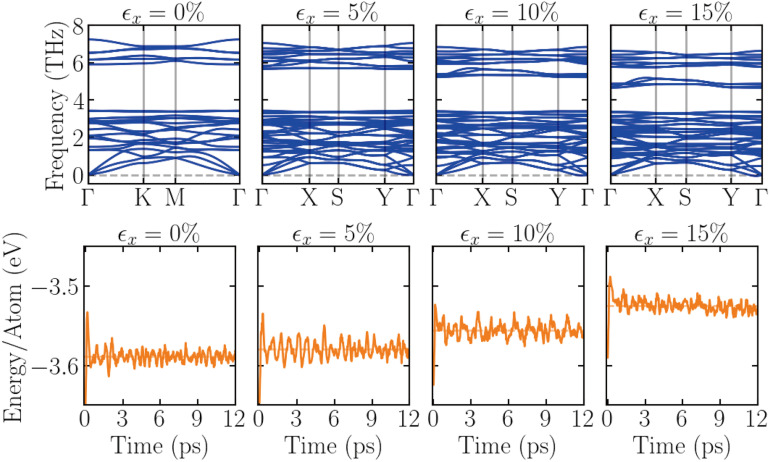
The frequency-dependent phonon dispersion from the phonon calculations (upper panels) and the dynamic evolution of the total energy per atom from the AIMD calculations (lower panels) for the CrI_3_ monolayer under different uniaxial strains, as indicated on the top of each panel.

CrX_3_ monolayers can withstand a large magnitude of uniaxial strain before their failure, as shown in Fig. 2(b). It is though important to assess and inspect the dynamical and thermal stability of these material when exploring their electronic properties under uniaxial strain, in particular, of large magnitude. To this end, Fig. 3 shows the phonon dispersion and the AIMD simulation results for the unstrained monolayer CrI_3_, as well as the structures under 5%, 10%, and 15% uniaxial strain applied along the zigzag (*x*) direction. As can be seen, the phonon dispersions indicate that the structure remains dynamically stable at 5% strain, while it becomes slightly unstable at 10%, with the emergence of imaginary (negative-frequency) phonon modes, narrowly localized near the *Γ* point on the right. As strain increases to 15%, the dynamical instability sets in as observed by the emergence of imaginary phonon modes, notably extended from the *Γ* point towards the *Y* point. The AIMD simulations further support this: the structure under 15% strain reveals that the atomic energies decrease, indicating the onset of the thermal instability. Based on these results, we have limited the applied strain to a maximum of 10% for the following discussion of the strain-dependent electronic properties.

**Fig. 4:**
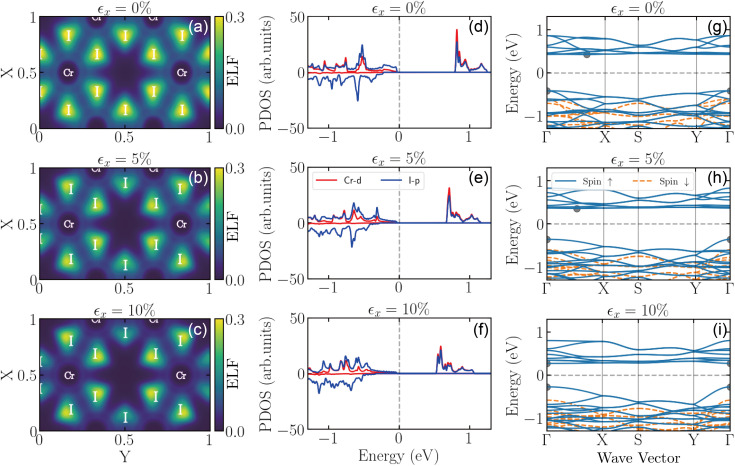
The electron localization function (a)–(c), the partial electronic density of states (d)–(f), and the electronic band structures (g)–(i) for the CrI_3_ monolayer under different uniaxial strains, as indicated at the top of each panel. In (a)–(c), the positions of the atoms are indicated with the respective element label, while the colors follow the color bars shown. In (d)–(e), the electronic density of states are provided for the two different spin-polarizations (see positive and negative contributions, respectively) and depict the different contributions of the d orbitals of the Cr atoms and the p orbitals of the I atoms. In (g)–(i), the electronic band structures around the Fermi energy are shown for the two different spin polarizations. Here, the spin-up states are represented by the blue solid lines, the spin-down states by the orange dashed lines, and the Fermi energy is represented by the gray dashed lines. Additionally, in (g) and (h), the conduction band minimum (CBM) and valence band maximum (VBM) are marked by gray solid dots.

In order to illustrate the strain-induced modifications in the bonding characteristics and electronic structure of the CrX_3_ (X = Cl, Br, I) monolayers under different levels of uniaxial strain applied along the zigzag (*x*) direction, Fig. 4 depicts the strain dependence of the electron localization function (ELF), the partial electronic density of states (PDOS), and the electronic band structure of the CrI_3_ monolayer as a representative of all three monolayers discussed here. In Fig. 4(a)–(c), the ELF maps depict how the electron localization evolves with increasing strain, providing insights into the re-distribution of the charge density around the Cr and I atoms. At zero strain in Fig. 4(a), the ELF shows localized electrons around the I atoms, with moderate charge density between the Cr and I atoms, indicating a relatively strong covalent bonding character. As the strain increases to 5% in Fig. 4(b), the ELF changes significantly, showing more localized electrons around the I atoms. The depletion of charge density between the Cr and I atoms suggests weakened bonding interactions as a result of charge density depletion. At 10% strain in Fig. 4(c), the electrons are even more localized around the I atoms. This behavior indicates a further weakening of the inter-atomic binding, which is consistent with the reduced mechanical strength under high strain.

The corresponding PDOS curves in Fig. 4(d)–(f) provide further insight into the evolution of the electronic structure with strain and the contribution of different electron orbitals. At zero strain in Fig. 4(d), the CrI_3_ monolayer exhibits a finite electronic band gap of 0.841 eV, with the highest valence states primarily composed of I-p orbitals and the lowest conduction states being dominated by both Cr-d and I-p orbitals. As strain increases to 10% in Fig. 4(e), an energy splitting of the Cr-d and I-p orbitals is induced, leading to a reduction in the electronic band gap to 0.544 eV. This suggests that uniaxial strain effectively tunes the electronic properties of CrI_3_ (and CrCl_3_, CrBr_3_) monolayers, making it promising for strain-tunable electronic applications.

The strain-induced electronic behavior observed in the PDOS results is also reflected in the corresponding electronic band structures shown in Fig. 4(g)–(i). As can be seen, with increasing strain, the electronic band structure exhibits a progressive reduction of the electronic band gap. Furthermore, an indirect-to-direct band-gap transition is also observed. It should be noted that we provide here the electronic band structures along the *k*-path respective of the rectangular symmetry of the strained structures in order to facilitate a consistent one-to-one comparison between the unstrained and strained conditions. We have, though, performed calculations along the *k*-path respective of the hexagonal symmetry for the unstrained material. We have carefully compared these different cases and confirm that beyond the quantitative differences in the bands along the *k*-path, the trends are very similar and the electronic band gap remains indirect with the same value of 0.841 eV. Our results also indicate similar qualitative trends for strain applied along the armchair (*y*) direction and for the other two monolayers (CrBr_3_ and CrCl_3_). However, quantitative differences are observed in the degree of electron localization, the charge depletion patterns, the energy splitting of atomic orbitals, and the indirect-to-direct transition of the electronic band gap.

## Conclusions

Using quantum-mechanical simulations, we have investigated the effect of uniaxial strain on CrX_3_ monolayers (with X = I, Br, Cl), focusing on their mechanical and electronic properties. This effect was compared for the strain along the two distinct strain directions: zigzag and armchair. We have unravelled this effect in the strain-dependent energetics, stress–strain relationships, electron localization functions, electronic density of states, and electronic band structures. The strain-dependent energetics showed that the magnetic ground state of CrX_3_ monolayers favors a ferromagnetic phase over a wide range of strain in both directions. At the same time, the stress–strain relationships exhibited a distinct anisotropy in the mechanical response of these monolayers. For the electronic properties of CrX_3_ monolayers, the application of strain induced changes in the inherent bonding characteristics, as mapped in the electron localization function. With increasing strain, an energy splitting of the electron orbitals in the electronic density of states and an indirect-to-direct transition of the band gap in the electronic band structure were observed. These observations provide a direct connection between strained CrX_3_ monolayers and the respective changes in their mechanical and electronic properties. The fundamental understanding provided through our analysis on the way uniaxial strain affects such materials is very insightful in designing novel applications, such as in the fields of strain-engineered electronics and spintronics, as well as underlines the expected modifications compared to the performance of ideal materials.

## Conflicts of interest

There are no conflicts to declare.

## Data Availability

The data supporting the findings of this study are available within the article. Additional datasets are available from the authors upon reasonable request, if applicable.
